# Scrub typhus point-of-care testing: A systematic review and meta-analysis

**DOI:** 10.1371/journal.pntd.0006330

**Published:** 2018-03-26

**Authors:** Kartika Saraswati, Nicholas P. J. Day, Mavuto Mukaka, Stuart D. Blacksell

**Affiliations:** 1 Mahidol-Oxford Tropical Medicine Research Unit, Faculty of Tropical Medicine, Mahidol University, Bangkok, Thailand; 2 Eijkman-Oxford Clinical Research Unit, Eijkman Institute for Molecular Biology, Jakarta, Indonesia; 3 Centre for Tropical Medicine & Global Health, Nuffield Department of Medicine, University of Oxford, Old Road Campus, Oxford, United Kingdom; Instituto Butantan, BRAZIL

## Abstract

**Background:**

Diagnosing scrub typhus clinically is difficult, hence laboratory tests play a very important role in diagnosis. As performing sophisticated laboratory tests in resource-limited settings is not feasible, accurate point-of-care testing (POCT) for scrub typhus diagnosis would be invaluable for patient diagnosis and management. Here we summarise the existing evidence on the accuracy of scrub typhus POCTs to inform clinical practitioners in resource-limited settings of their diagnostic value.

**Methodology/principal findings:**

Studies on POCTs which can be feasibly deployed in primary health care or outpatient settings were included. Thirty-one studies were identified through PubMed and manual searches of reference lists. The quality of the studies was assessed with the Quality Assessment of Diagnostic Accuracy Studies 2 (QUADAS-2). About half (n = 14/31) of the included studies were of moderate quality. Meta-analysis showed the pooled sensitivity and specificity of commercially available immunochromatographic tests (ICTs) were 66.0% (95% CI 0.37–0.86) and 92.0% (95% CI 0.83–0.97), respectively. There was a significant and high degree of heterogeneity between the studies (I^2^ value = 97.48%, 95% CI 96.71–98.24 for sensitivity and I^2^ value = 98.17%, 95% CI 97.67–98.67 for specificity). Significant heterogeneity was observed for total number of samples between studies (p = 0.01), study design (whether using case-control design or not, p = 0.01), blinding during index test interpretation (p = 0.02), and QUADAS-2 score (p = 0.01).

**Conclusions/significance:**

There was significant heterogeneity between the scrub typhus POCT diagnostic accuracy studies examined. Overall, the commercially available scrub typhus ICTs demonstrated better performance when ‘ruling in’ the diagnosis. There is a need for standardised methods and reporting of diagnostic accuracy to decrease between-study heterogeneity and increase comparability among study results, as well as development of an affordable and accurate antigen-based POCT to tackle the inherent weaknesses associated with serological testing.

## Author summary

Scrub typhus is a disease caused by a mite-transmitted bacterium, *Orientia tsutsugamushi*. Aside from fever and necrotic skin lesion at the site of mite inoculation (eschar), scrub typhus has virtually no distinctive clinical features, making diagnosis without laboratory tests difficult. However, the laboratory tests often have limited diagnostic accuracy and are generally in limited supply in resource-limited or outpatient settings. In those settings, point-of-care testing (POCT) can be useful in making a quick diagnosis. In this study, the authors reviewed the available evidence on the diagnostic value of scrub typhus POCT. A meta-analysis, focusing on commercially available immunochromatography tests (ICTs), was done. The pooled sensitivity and specificity were 66.0% (95% CI 0.37–0.86) and 92.0% (95% CI 0.83–0.97), respectively. This indicates better performance when ‘ruling in’ a positive diagnosis rather than ‘ruling out’ a negative diagnosis. As ICTs have low false positive rates, confidence in interpreting a positive result can be high. The studies reviewed displayed wide variation in both methodology and quality, indicating a need to develop standards for study methodology and reporting.

## Introduction

Scrub typhus is a febrile illness caused by the obligate intracellular bacterium, *Orientia tsutsugamushi*. It is transmitted by the bite of infected larvae of a number of trombiculid mite species known to be prevalent in Asia, the Pacific Rim islands, pockets in the north of Australia, and some areas of Chile [1–4]. In 2010, a novel species from the same genus, *Orientia chuto* sp. nov., was identified in an acutely febrile patient infected in Dubai [5]. Scrub typhus responds to certain antibiotics (i.e. doxycycline, tetracycline, azithromycin, chloramphenicol), but if left untreated, the mortality rate may reach 70% [6]. One estimate, based on scant data, is that there are one billion people at risk of this disease; with one million clinical cases annually in Southeast Asia alone [1]. Although the exact prevalence of scrub typhus is not available, several studies showed that the disease burden in rural Asia is high–causing in some areas over 20.0% of febrile illness admitted to hospital [7,8].

Infected patients usually present with acute fever; lymphadenopathy (regional or generalised) and sensorineural hearing loss may occur, neither of which is sensitive or specific enough for establishing diagnosis [1]. With few distinguishing clinical characteristics, scrub typhus is difficult to differentiate from other tropical febrile illnesses, such as dengue, typhoid fever, leptospirosis, and murine typhus [1,9]. The presence of the pathognomonic eschar, the painless black crust at the site of mite inoculation, can help in establishing clinical diagnosis due to its high specificity (98.9%), however, its presence in patients varies widely (7.0%-97.0%) [1,10–12].

Therefore, the role of laboratory tests in establishing diagnosis in scrub typhus cases is very important. Laboratory tests for scrub typhus often have limited diagnostic accuracy and are generally in limited supply in resource-limited or outpatient settings [9]. Failure in diagnosing scrub typhus may result in prolonged illness, complications including pneumonitis, acute respiratory distress syndrome, renal failure, meningoencephalitis, and unnecessary treatment with inappropriate antibiotics [1,9,13].

Serology remains the mainstay of diagnosis. The immunofluorescent assay (IFA) and immunoperoxidase test (IIP) are considered imperfect gold standards, in view of their limitations which include high expense, requirement for substantial training to perform, inter-operator variability in result interpretation, and the often-retrospective nature of diagnosis that does not help in directing treatment [1,9,12,14,15]. Another antibody detection method, the enzyme-linked immunosorbent assay (ELISA) has been developed and shown to have both sensitivity and specificity of greater than 90.0%; however this is highly dependent on endemicity and the application of a previously investigated and geographically-based cut-off [1,16]. Besides antibody-based diagnostics, molecular detection methods, including using the polymerase chain reaction (PCR) to detect various genes targets (e.g. 47 kDa, 56 kDa, *groEL*, 16S rRNA genes) have also been developed, however they have limitations in terms of diagnostic sensitivity due to the limited period of rickettsaemia [1,9,12,14]. PCR is still deemed impractical in resource-limited endemic areas because it requires considerable training and expense [1,9,12,14]. The bacteria can be isolated through *in vitro* and *in vivo* cultivation methods, such as cell culture and mouse inoculation, respectively [1,9]. These methods need considerable training, biosafety level 3 (BSL 3) laboratory containment facilities for large-scale propagation, and usually take several weeks which contributed to the retrospective nature of the diagnosis [1,9].

Therefore, there is clearly a need for affordable point-of-care testing (POCT) for scrub typhus diagnosis in endemic settings with resource constraints. There are varied definitions of POCT, but fundamentally POCT should provide quick results to inform patient management and be convenient enough to be performed close to the patient (i.e. not in a central laboratory) [17,18].

Immunochromatographic tests (ICTs), dot-blot, and loop-mediated isothermal amplification (LAMP) assays all have the principal qualities of POCT. ICTs and dot-blot tests have the same inherent problems of IFA as serology-based tests (e.g. the retrospective nature of diagnosis in cases where diagnosis relies on a convalescent sample, delicate cut-off setting), while offering more simplicity and speed [1,9]. LAMP is an alternative technique which involves amplification and detection of bacterial DNA. Similar in principle to conventional PCR assays, LAMP assay does not require intricate DNA extraction, a thermocycler, or special equipment to read the result [9,19,20].

This study aims to summarise the existing evidence on the accuracy of scrub typhus POCTs to inform clinical practitioners of their diagnostic value when providing care in resource-limited settings where scrub typhus is endemic.

## Methods

### Eligibility criteria

This review included articles on POCTs that would be feasible in primary health care provider or outpatient settings. Only articles published in English were included. To ensure feasibility in resource-limited settings, studies evaluating methods which were inherently more complicated, requiring relatively high levels of expertise and/or specialised equipment were excluded. Articles on POCTs not performed on human samples were excluded. Studies on the Weil-Felix test were excluded due to its established poor diagnostic accuracy and the lengthy time required to perform [9,21]. Meta-analysis and meta-regression were performed on studies of commercially available POCTs with an extractable diagnostic accuracy 2 by 2 table.

### Search strategy

After a preliminary search, ICT, dot-blot, and LAMP were searched for specifically. The search was conducted on articles cited in PubMed up to 2 February 2017 combining the search terms ‘scrub typhus’, ‘immunochromatography’, ‘dot blot immunoassay’, and ‘loop mediated isothermal amplification’ without any other restrictions (i.e., "scrub typhus" AND (rapid diagnosis OR immunochromatograph* OR dot blot immunoassay OR loop mediated isothermal amplification). The titles and abstracts were screened and the full text of relevant articles were reviewed. Manual screening of the reference list of relevant articles was also performed.

### Quality assessment

The quality of the studies was assessed with the Quality Assessment of Diagnostic Accuracy Studies 2 (QUADAS-2) [22]. QUADAS-2 was used as a quality scoring system to determine the risk of bias and the applicability of the paper [22]. It evaluates four main areas: ‘patient selection’, ‘index test’, ‘reference standard’, and ‘flow and timing’ [22]. These are assessed by using seven ‘signalling questions’ (e.g., “was a case-control design avoided?”) with ‘yes’, ‘no’, and ‘unclear’ answer [22]. The answers to these ‘signalling questions’ were then used to judge whether the risk of bias is low and if there is low concern for the applicability of the research [22]. If the response to the risk of bias and applicability questions were ‘low risk’ or ‘low concern’, the articles were given one point each. The articles were grouped based on their score into high (6–7 points), moderate (4–5 points), and low (0–3 points) quality categories.

### Data extraction

Data was extracted primarily by one author (KS) and where the results were unclear a second author (SB) was consulted. The data was recorded on a form developed through an iterative process to ensure that all the required data could be collected for future reference. The parameters extracted include: citation information, methodology (i.e., study design, participant characteristics, index and reference test details), and the diagnostic accuracy results (including sensitivity, specificity, positive predictive value (PPV), negative predictive value (NPV), and if available, numbers required to construct a 2 by 2 contingency table.

### Statistical data analysis and reporting

The extracted data were compiled into summary tables and analysed through narrative synthesis. Meta-analysis and meta-regression were performed on commercially available POCT diagnostic accuracy data, excluding studies with low quality (i.e. QUADAS-2 score of 3 or less). The ones in development stage/prototype were not included in the meta-analysis and meta-regression, but included in the narrative synthesis. If one study derived more than one 2 by 2 table, each table was extracted as separate data. However, if one study used more than one reference test cut-off titre, only data using one cut-off value above 1:3,200 were used to ensure accuracy [12]. In performing the meta-regression, relevant signalling questions with ‘unclear’ as the answers were entered as ‘no’ to turn these into dichotomous variables. Statistical analysis was done with STATA/IC 14.0 (College Station, TX) using MIDAS and METANDI commands. In the meta-analyses, heterogeneity was assessed using the Chi-square statistic, higher values of the Chi-square (and hence low p-value) being consistent with heterogeneity. In the summary statistics of the resulting forest plot, overall sensitivity and specificity were estimated and reported alongside the 95% confidence interval. In addition, study specific estimates were provided in the same plot to visualize how the estimates from each of the studies deviate from the overall estimate. Most of the results was presented graphically. The data was analysed, summarised, and presented following the Preferred Reporting Items for Systematic reviews and Meta-Analyses (PRISMA) statement as much as possible [23]. This review was registered in the International Prospective Register for Systematic Review (PROSPERO) with registration number CRD42017056727.

## Results

### Search results

**Fig 1:**
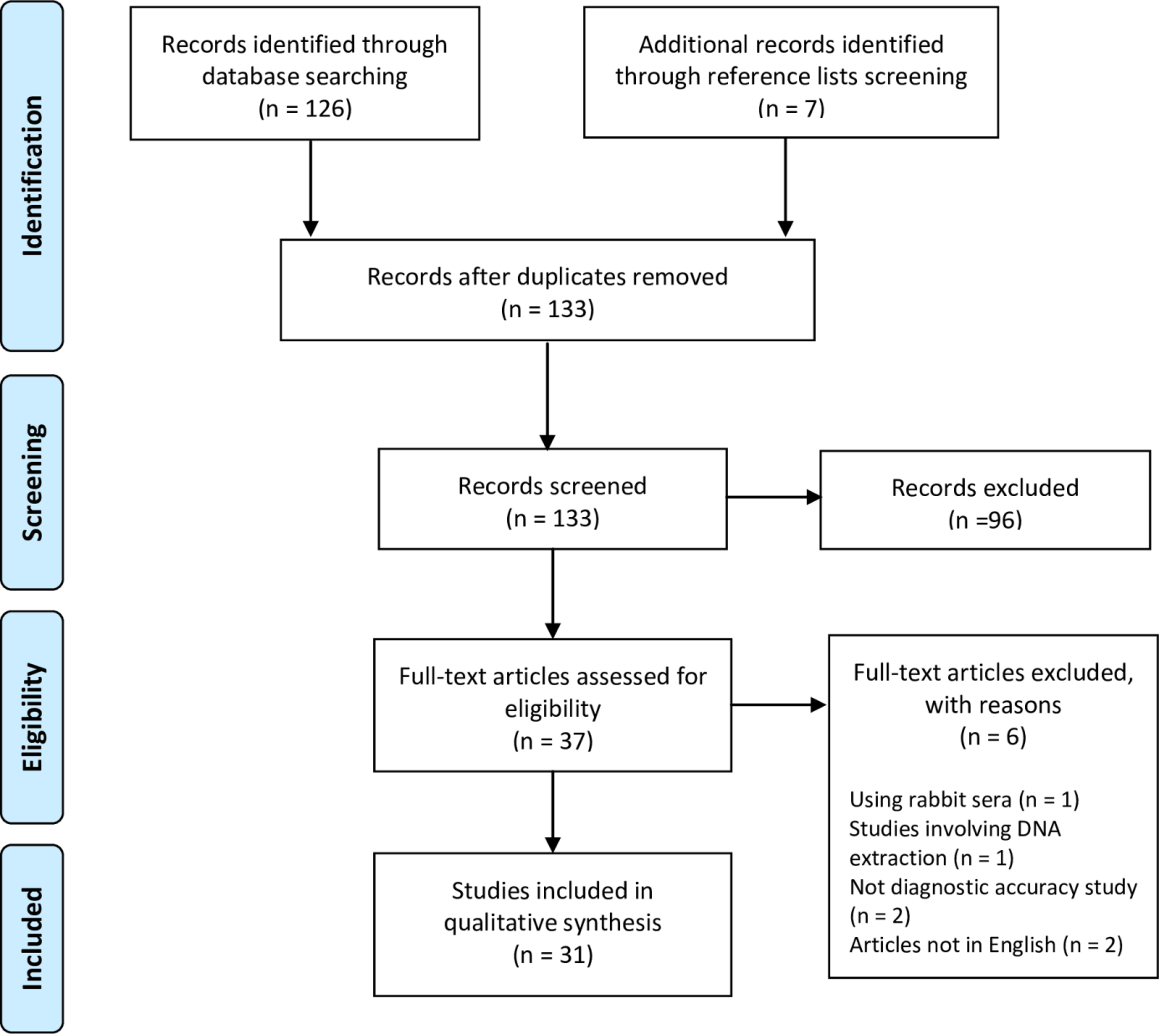
PRISMA flow diagram.

There were 133 articles in total identified through database searching and reference list screening (Fig 1). After title and abstract screening and full text review, we included 31 relevant articles. There were six articles excluded after full text review. Since this study only focused on human diagnostics, one article using rabbit sera in its negative sera panel was excluded. One study involved DNA extraction, which is not applicable as a POCT in resource-limited settings. Four other papers were excluded due to language (not in English, n = 2) and study design (not experimental diagnostic accuracy studies, n = 2). There were 20 articles on ICTs, eight articles on dot-blot assays, four articles on LAMP assays, and one each on a passive hemagglutination assay, an IgM dot immunobinding assay, and a latex agglutination test. Four articles evaluated more than one type of diagnostic test. There were 11 studies that evaluated diagnostic tests still in development and 21 studies on prototype/commercial tests.

### Characteristics of the included studies

In total, there were 6,772 samples analysed. The samples were taken from 12 countries, with most studies recruiting in Thailand (n = 15, 48.4% of included studies), India (n = 5, 16.1%), Laos (n = 3, 9.7%), and Korea (n = 3, 9.7%). There was one study each (3.2%) conducted on samples from Sri Lanka, Nepal, Malaysia, Peru, Indonesia, United States of America, and Australia. There were five studies (16.1%) with unclear sample collection location. Ten (32.2%) studies collected paired samples (acute and convalescent phase), while 15 studies (48.4%) did not provide sufficient details on sample collection timing (Table 1).

**Table 1:** Summary of included studies.

First author	Year	Sample collection location	Index test assay type	Reference test assay type	QUADAS a 2 score
Anitharaj et al [33]	2016	India	ICT IgM	ELISA	5
Blacksell et al [34]	2010	Laos	ICT IgM	IFA	6
Blacksell et al [28]	2010	Laos and Thailand	ICT IgM; ICT IgG, IgM	IFA and/or PCR and/or culture	5
Blacksell et al [19]	2012	Thailand	ICT IgM; ICT IgG, IgM; LAMP	STIC (cell culture isolation, PCR, IFA)	6
Cao et al [25]	2007	China	ICT IgG, IgM; ICT IgG; ICT IgM	Unclear	2
Ching et al [24]	2001	Unclear	ICT IgG, IgM; ICT IgG; ICT IgM	IFA	2
Chinprasatsak et al [35]	2001	Thailand	Dot-blot IgG, IgM	IIP	6
Coleman et al [36]	2002	Thailand	Dot-blot IgG, dot-blot IgM, ICT IgG, ICT IgM	IIP	7
Huber et al [37]	2012	Unclear	LAMP	PCR	2
Kim et al [38]	1993	Unclear	Passive hemagglutination assay	IFA	3
Kim et al [39]	2013	Korea	ICT IgG, IgM; dot-blot IgG	Unclear	2
Kim et al [40]	2016	Korea, Sri Lanka, India	ICT IgG, IgM	IFA	3
Kingston et al [41]	2015	Thailand, Nepal	ICT IgM PAb, Mab	IFA	6
Koay et al [42]	1995	Unclear	IgM dot-immunobinding assay	IIP	3
Lee et al [27]	2014	Korea	ICT IgG, IgM, IgA	IFA	4
Paris et al [43]	2008	Thailand, Laos	LAMP	In vitro isolates, IFA, PCR, ICT IgG & IgM	3
Paris et al [20]	2011	Thailand	ICT IgM, LAMP	STIC (Cell culture isolation, PCR, IFA)	6
Pradutkanchana et al [44]	1997	Thailand	Dot-blot IgG, IgM	IFA	4
Prakash et al [45]	2006	India	Dot-blot IgG, IgM	Dot EIA, ELISA, clinical response to antibiotics^a^	3
Ramyasree et al [46]	2015	Unclear	ICT IgM	ELISA	5
Rodkvamtook et al [47]	2015	Thailand	Dot-blot IgG, IgM	IFA	5
Silpasakorn et al [48]	2012	Thailand	ICT IgM, ICT IgG	PCR & IFA	4
Silpasakorn et al [49]	2012	Thailand	ICT IgG, IgM, IgA	PCR & IFA	3
Stephen et al [50]	2015	India	ICT IgG, IgM, IgA	ELISA IgM, IgG	5
Stephen et al [51]	2016	India	ICT IgG, ICT IgM	IFA	5
Watt et al [52]	1998	Thailand	Dot-blot IgG, IgM	IFA	5
Watthanaworawit et al [29]	2015	Thailand	ICT IgM; IgG, IgM, IgA	PCR & IFA	5
Weddle et al [53]	1995	Malaysia, Peru, Indonesia, USA	Dot-blot immunoassay IgG, IgM	IFA	4
Wilkinson et al [54]	2003	Thailand, Australia	ICT IgG, ICT IgM	Unclear	2
Wongchotigul et al [55]	2005	Thailand	Latex agglutination test	IFA	4
Zhang et al [26]	2011	China	InBios ICT IgG, ICT IgM	IgM and IgG antibodies detection (tests unclear) and PCR	4

^a^Doxycycline or chloramphenicol

### Quality of articles

**Fig 2:**
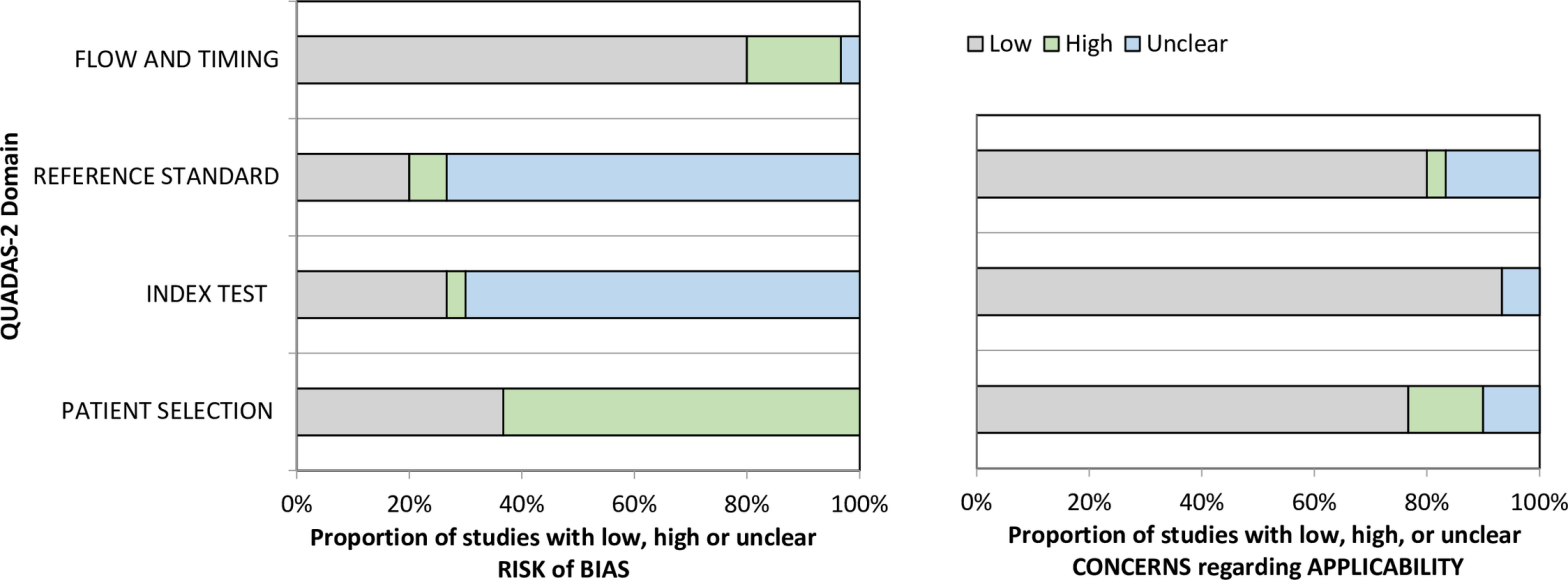
QUADAS-2 finding per domain.

There are 7, 14, and 10 articles with good, moderate, and low quality, respectively (Table 1). Two articles fulfilled all of the QUADAS-2 main criteria (S1 Dataset). Most of the articles (n = 8, 25.8%) scored 5 points. Two articles (6.5%) scored full points for the risk of bias component (i.e. 4 points), although most of the articles scored only 1 point (n = 12, 38.7%). The majority of the articles scored 3 points in the applicability component (n = 22, 71.0%). There were 24.4% (n = 53/217) sub-questions with ‘unclear’ response. Among the ‘unclear’ response, most of those responses (n = 45, 84.9%) were observed in the index test and reference standard domain of the risk of bias assessment (Fig 2). The main reason for this is that most of the articles did not mention explicitly whether they performed blinding during the conduct and interpretation of both the index test (n = 23, 74.2%) and reference test (n = 25, 80.6%) (S2 Dataset). There were 20 studies (64.5%) with a case-control study design.

### Performance of POCT

#### IgM ICT

**Fig 3:**
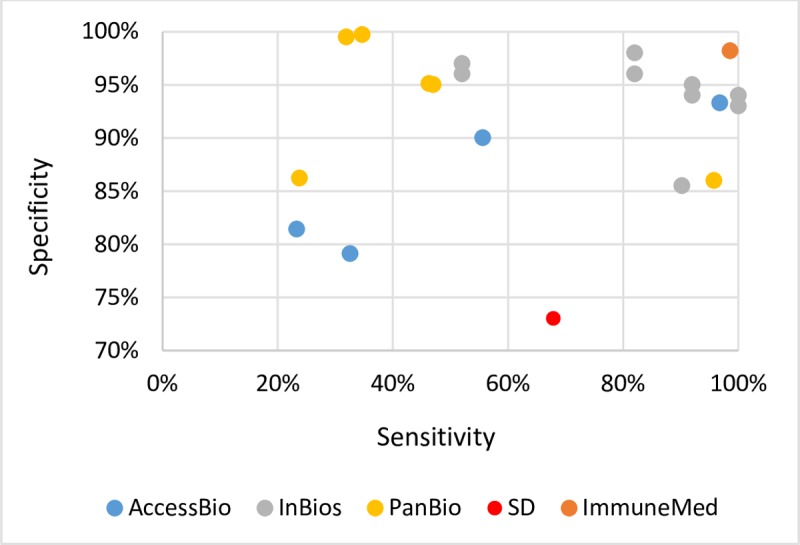
IgM ICT sensitivity and specificity range*.

There were five manufacturers of IgM ICT identified (Table 2), namely: AccessBio, InBios, PanBio, Standard Diagnostic (SD), and ImmuneMed. There were two studies (6.6%) that assessed in-house tests [24,25]. The sensitivity ranged from 23.3% to 100.0%, and the specificity ranged from 73.0% to 100.0% (Fig 3, results from Zhang et al, 2011 and Lee et al, 2014 were not plotted since only sensitivity values were presented [26,27]). The accuracy across data points of the same manufacturers varied across the studies. The InBios IgM ICT tests reported >80.0% sensitivity and >90.0% specificity on average. The ImmuneMed IgM ICT demonstrated sensitivity of 99.0% and specificity of 98.0%, however, there was only one data point for this manufacturer. One of the studies reported sensitivity and specificity for the AccessBio ICT IgM of 97.0% and 93.0%, respectively. However, the other AccessBio studies did not demonstrate such a high degree of accuracy (Fig 3).

**Table 2:** Summary of ICT IgM.

Assay	Study	Year	Location of sample collection	Sample collection timing	Reference test assay type	Reference assay positivity cut-off	Sensitivity (%)/ specificity (%)
CareStart, AccessBio	Blacksell et al [28]	2010	Laos and Thailand	Acute phase	IFA and/or PCR and/or culture	IFA: 4-fold rise in paired serum samples	96.8/93.3
	Blacksell et al [19]	2012	Thailand	Acute (median 5 days of fever, IQR 3 to 7 days) and convalescent phase	STIC	IFA: an admission IgM titre of 1:12,800, and/or a 4-fold rising IgM titre in paired serum samples	55.6/90.0
	Watthanaworawit et al [29]	2015	Thailand	Acute (median 2 days of fever, IQR: 2–3 days) and convalescent phase (median interval to convalescent sample collection was 14 days, range: 11–30 days).	PCR & IFA	≥ 4-fold increase in IFA IgM titer, 1:25,600	Acute samples: 23.3/81.4
							Paired samples: 32.6/79.1
ImmuneMed	Stephen et al [51]	2016	India	Acute phase with partly paired samples	IFA	1:40	87.0/94.6
InBios	Zhang et al [26]	2011	China	Acute phase: 2 to 10 days after onset	IgM and IgG antibodies detection (unclear) and PCR	Unclear	Sensitivity 93.9%
	Silpasakorn et al [48]	2012	Thailand	Acute and convalescent phase	PCR & IFA	IFA: IgM 1:400 or a 4-fold increase	90.2/85.5
InBios prototype	Kingston et al [41]	2015	Thailand, Nepal	Unclear	IFA	1:400	PAb: 52.0/97.0
						1:400	MAb: 52.0/96.0
						1:1,600	PAb: 82.0/98.0
						1:1,600	MAb: 82.0/96.0
						1:6,400	PAb: 92.0/95.0
						1:6,400	MAb: 92.0/94.0
						1:25,600	PAb: 100.0/94.0
						1:25,600	MAb: 100.0/93.0
PanBio	Blacksell et al [34]	2010	Laos	Admission phase: median 6 days of fever (IQR = 5–10 days); median interval to convalescent sampling: 7 days (IQR = 5–10 days)	IFA	≥ 1:400	23.8/86.2
						4-fold increase in paired samples	31.9/99.5
						Combination of the above	34.7/99.7
	Paris et al [20]	2011	Thailand	Mostly paired samples. Median fever before admission = 5 days (IQR: 3–7)	STIC	IFA: an admission IgM titer ≥1:12,800 and/or a 4-fold increase	47.0/95.0
	Blacksell et al [19]	2012	Thailand	Acute (median 5 days of fever, IQR 3 to 7 days) and convalescent phase	STIC	IFA: an admission IgM titer ≥1:12,800 and/or a 4-fold increase	46.3/95.1
PanBio prototype	Wilkinson et al [54]	2003	Thailand, Australia	Unclear	Unclear	Unclear	95.8/86.0
SD Bioline Tsutsugamushi	Blacksell et al [19]	2012	Thailand	Acute (median 5 days of fever, IQR 3 to 7 days) and convalescent phase	STIC	IFA: an admission IgM titer ≥1:12,800 and/or a 4-fold increase	67.9/73.0
	Ramyasree et al [46]	2015	India?	Unclear	ELISA	Unclear	Agreement 97%
In-house tests	Ching et al [24]	2001	Unclear	Unclear	IFA	>1:40	74.0/99.0
	Cao et al [25]	2007	China	Unclear	Unclear	Unclear	81.2%/100.0

#### Total antibody ICT

**Fig 4:**
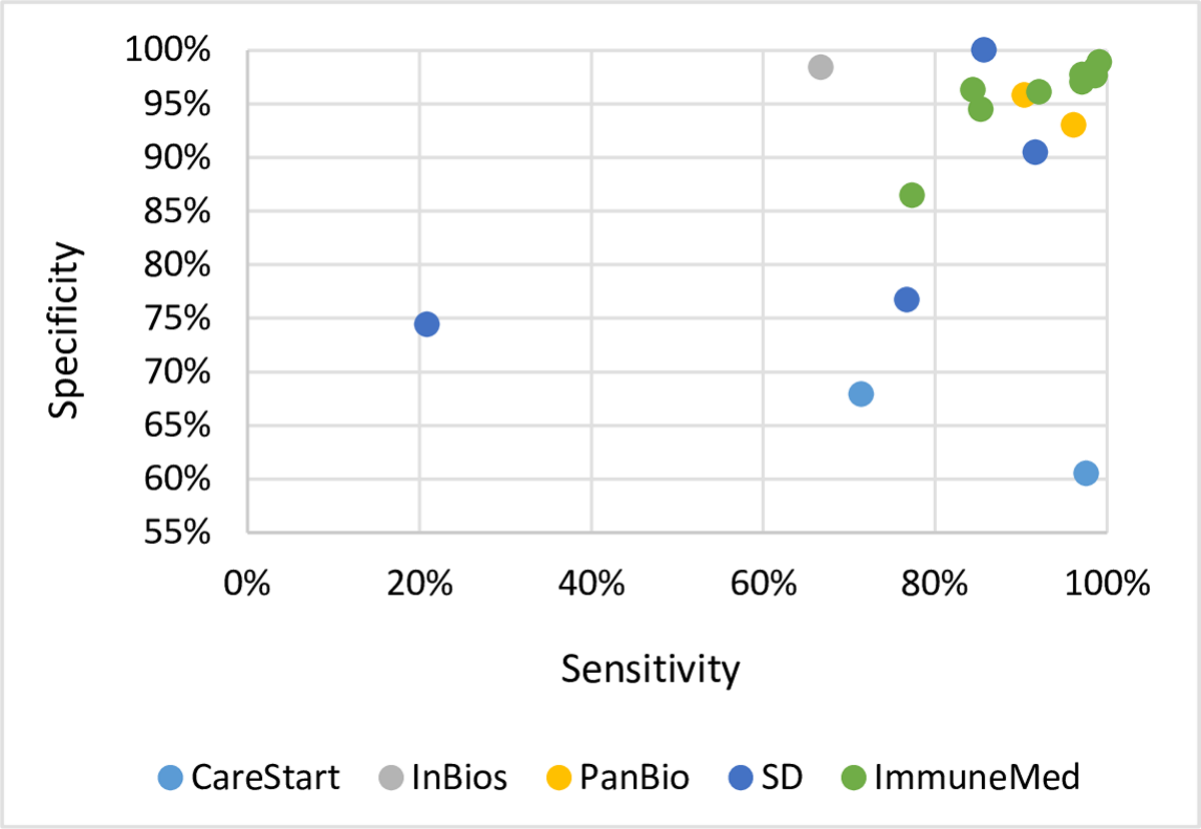
Total antibody ICT sensitivity and specificity range*.

The ICTs with IgG, combination of IgG and IgM, and combination of IgG, IgM, and IgA as the detection target were grouped together under ‘total antibody ICT’ (Table 3). There were five manufacturers identified, namely: AccessBio, ImmuneMed, InBios, PanBio, and SD. The remaining studies (n = 3, 6.6%) assessed the diagnostic performance of in-house tests in development. The sensitivity and specificity ranged from 20.9% to 99.1% and 67.9% to 100.0%, respectively (Fig 4, results from and Zhang et al, 2011 and Lee et al, 2014 were not plotted since only sensitivity values were presented [26,27]). As in the case of IgM ICT, the accuracy across data points of total antibody ICT of the same manufacturer varies. The ImmuneMed total antibody ICT demonstrated >95.0% specificity and >80.0% sensitivity.

**Table 3:** Summary table of total antibody ICT.

Assay	Study	Year	Location of sample collection	Sample collection timing	Reference test assay type	Reference assay positivity cut-off	Sensitivity (%)/ specificity (%)
CareStart, AccessBio	Blacksell et al^a^ [28]	2010	Laos and Thailand	Acute phase	IFA and/or PCR and/or culture	IFA: 4-fold rise in paired serum samples	97.6/71.4
	Blacksell et al^a^ [19]	2012	Thailand	Acute (median 5 days of fever, IQR 3 to 7 days) and convalescent phase	STIC	IFA: an admission IgM titre of 1:12,800, and/or a 4-fold rising IgM titre in paired serum samples	60.5/67.9
ImmuneMed	Kim et al^a^ [39]	2013	Korea	Unclear	Unclear	Unclear	99.1/98.9
	Kim et al^b^ [40]	2016	Korea^d^	Unclear	IFA IgM	1:10	98.6/98.2
					IFA IgG	1:40	97.1/97.7
			Korea^e^	Unclear	IFA IgM	1:10	98.6/97.6
					IFA IgG	1:40	97.1/97.0
			Sri Lanka	Unclear	IFA	Unclear	92.1/96.1
			India	Unclear	IFA IgM	1:10	Sensitivity 86.0%
					IFA IgG	1:40	Sensitivity 92.0%
	Stephen et al^b^ [51]	2016	India	Acute and convalescent phase	IFA	1:80	77.3/86.4
InBios	Zhang et al^b^ [26]	2011	China	Acute phase: 2 to 10 days after onset of illness	IgM and IgG antibodies detection (unclear) and PCR	Unclear	Sensitivity 90.9%
	Silpasakorn et al^c^ [48]	2012	Thailand	Acute and convalescent phase	PCR & IFA	IFA: IgG 1:800 or a 4-fold increase	66.7%/98.4
PanBio	Coleman et al^b^ [36]	2002	Thailand	Acute phase	IIP	1:400	90.4/95.8
Panbio prototype	Wilkinson et al^b^ [54]	2003	Thailand, Australia	Unclear	Unclear	Unclear	96.1/93.0
SD Bioline Tsutsugamushi	Silpasakorn et al^c^ [49]	2012	Thailand	Acute phase: median 6 days of fever (range 1–47 days); median interval between obtaining admission and convalescence phase: 13 days (range 3–32 days)	PCR & IFA	IFA: IgM or IgG IFA assay titre > 1:400 or a 4-fold increase	66.7/98.4
	Lee et al^c^ [27]	2014	Korea	Unclear	IFA	4-fold rise or single titre ≥ 1:160	Sensitivity 72.6%
	Stephen et al^c^ [50]	2015	India	Acute and convalescence phase	ELISA IgM	Unclear	91.7/90.5
					ELISA IgG	Unclear	85.7/100.0
	Watthanaworawit et al^c^ [29]	2015	Thailand	Median fever = 2 days (IQR: 2–3 days), and the median interval between obtaining initial acute-phase specimens and convalescent specimens was 14 days (range: 11–30 days).	PCR & IFA	≥ 4-fold increase in IFA IgM titre, 1:25,600	Acute samples: 20.9/74.4
							Paired samples: 76.7/76.7
In-house tests	Ching et al^a^ [24]	2001	Unclear	Unclear	IFA	>1:40	IgG: 86.0/97.0
							IgG, IgM: 89.0/97.0
	Cao et al^a^ [25]	2007	China	Unclear	Unclear	Unclear	IgG: 94.6/98.9
							IgG, IgM: 98.2/98.1

^a^Total antibody: IgG & IgM

^b^IgG

^c^IgG, IgM, IgA

^d^Healthy control sera

^e^Diseases non-scrub typhus control sera

#### Dot-blot

Aside from the in-house tests assessed by four studies, there were two dot-blot assay manufacturers, namely Integrated Diagnostics and PanBio. The range was 59.6% to 100.0% and 83.0% to 98.7% for sensitivity and specificity, respectively (Table 4).

**Table 4:** Summary of articles on dot-blot, LAMP and other assays.

Assay type	Assay	Study	Year	Location of sample collection	Sample collection timing	Reference test assay type	Reference assay positivity cut-off	Sensitivity (%)/ specificity (%)
Immunoblot	Dip-S-Ticks, Integrated Diagnostics	Pradutkanchana et al [44]	1997	Thailand	Unclear	IFA	≥1:400 or a 4-fold or greater rise in IFA titre to at least 1:200	94.0/98.7
		Chinprasatsak et al [35]	2001	Thailand	Acute and convalescent phase	IIP	“Four-fold or greater rise in IIP titre to at least 1:200, or a single IgM antibody titre of ≥ 1:400 and/or IgG antibody titre of ≥ 1:1,600.”	86.7/94.3
		Coleman et al [36]	2002	Thailand	Acute phase	IIP	1:400	IgM: 60.3/97.4 IgG: 59.6/95.3
	Rickettsia Screen, PanBio	Prakash et al [45]	2006	India	Acute phase	Dot EIA, ELISA, clinical response to antibiotics*	ELISA: ≥16 PanBio units	100.0/93.5
	In-house	Weddle et al [53]	1995	Malaysia, Peru, Indonesia, USA	Acute phase, unclear for negative panel	IFA	<1:64, >1:128, if in between depends on clinical condition	90.0/83.0
		Watt et al [52]	1998	Thailand	High-antigen dipstick: median = 4 days of fever (range = 2–14)	IFA	IgG 1:1600 and/or IgM 1:400	67.0/98.0
					Low-antigen dipstick: median = 4 days of fever (range = 2–30)			100.0/98.0
		Kim et al^ [39]	2013	Korea	Unclear	Unclear	Unclear	97.7/98.6
		Rodkvamtook et al [47]	2015	Thailand	Acute phase: fever no more than 2 weeks;	IFA	1:400 or 4-fold increase for IgG & IgM	98.5/96.3
					Paired sample. Convalescent phase: collected after 3–14 days after the acute sample.			98.3/97.0
LAMP	Loopamp kit, Eiken Chemical LAMP	Paris et al [43]	2008	Thailand, Laos	Acute phase for positive panel	IFA	4-fold rise or single titre ≥ 1:160	Sensitivity 72.6%
		Paris et al [20]	2011	Thailand	Mostly paired samples. Median fever before admission = 5 days (IQR: 3–7)	In vitro isolates, IFA, PCR, ICT IgG & IgM	1:400 and four-fold rise of paired serum for IgM IFA	100.0/100.0
	In-house LAMP	Huber et al [37]	2012	Not stated	Not stated in detail	PCR	N/A	90.0/80.0
	Combined Panbio ICT IgM and in-house LAMP	Blacksell et al [19]	2012	Thailand	Acute (median 5 days of fever, IQR 3 to 7 days) and convalescent phase	STIC (cell culture isolation, PCR, IFA)	IFA: an admission IgM titre of 1:12,800, and/or a 4-fold rising IgM titre in paired samples	66.7/90.6
	Combined SD ICT IgM and in-house LAMP	Blacksell et al [19]	2012	Thailand	Acute (median 5 days of fever, IQR 3 to 7 days) and convalescent phase	STIC (cell culture isolation, PCR, IFA)	IFA: an admission IgM titre of 1:12,800, and/or a 4-fold rising IgM titre in paired samples	77.2/68.2
	Combined AccessBio ICT IgG, IgM and in-house LAMP	Blacksell et al [19]	2012	Thailand	Acute (median 5 days of fever, IQR 3 to 7 days) and convalescent phase	STIC (cell culture isolation, PCR, IFA)	IFA: an admission IgM titre of 1:12,800, and/or a 4-fold rising IgM titre in paired samples	68.5/84.9
	Combined AccessBio ICT IgM and in-house LAMP	Blacksell et al [19]	2012	Thailand	Acute (median 5 days of fever, IQR 3 to 7 days) and convalescent phase	STIC (cell culture isolation, PCR, IFA)	IFA: an admission IgM titre of 1:12,800, and/or a 4-fold rising IgM titre in paired samples	71.6/63.2
Others	Passive hemagglutination assay	Kim et al [38]	1993	Unclear	Unclear	IFA	Seroconversion or 4-fold rise	99.0/98.9
	IgM dot-immunobinding assay	Koay et al [42]	1995	Unclear	Unclear	IIP	1:50	90.4/81.4
	Latex agglutination assay	Wongchotigul et al [55]	2005	Thailand	Unclear	IFA	1:400	89.1/98.2

*Doxycycline or chloramphenicol

^IgG only

#### LAMP

The Loopamp LAMP kit (Eiken Chemical, Japan) was assessed in two studies (Table 4). The other two studies evaluated in-house tests. The sensitivity ranged from 66.7% to 100.0% and specificity ranged from 63.2% to 100.0%.

#### Other methods

There was one study each on a passive hemagglutination assay, an IgM dot-immunobinding assay, and a latex agglutination assay (Table 4). These tests were all in the development phase.

### Meta-analysis results

**Fig 5:**
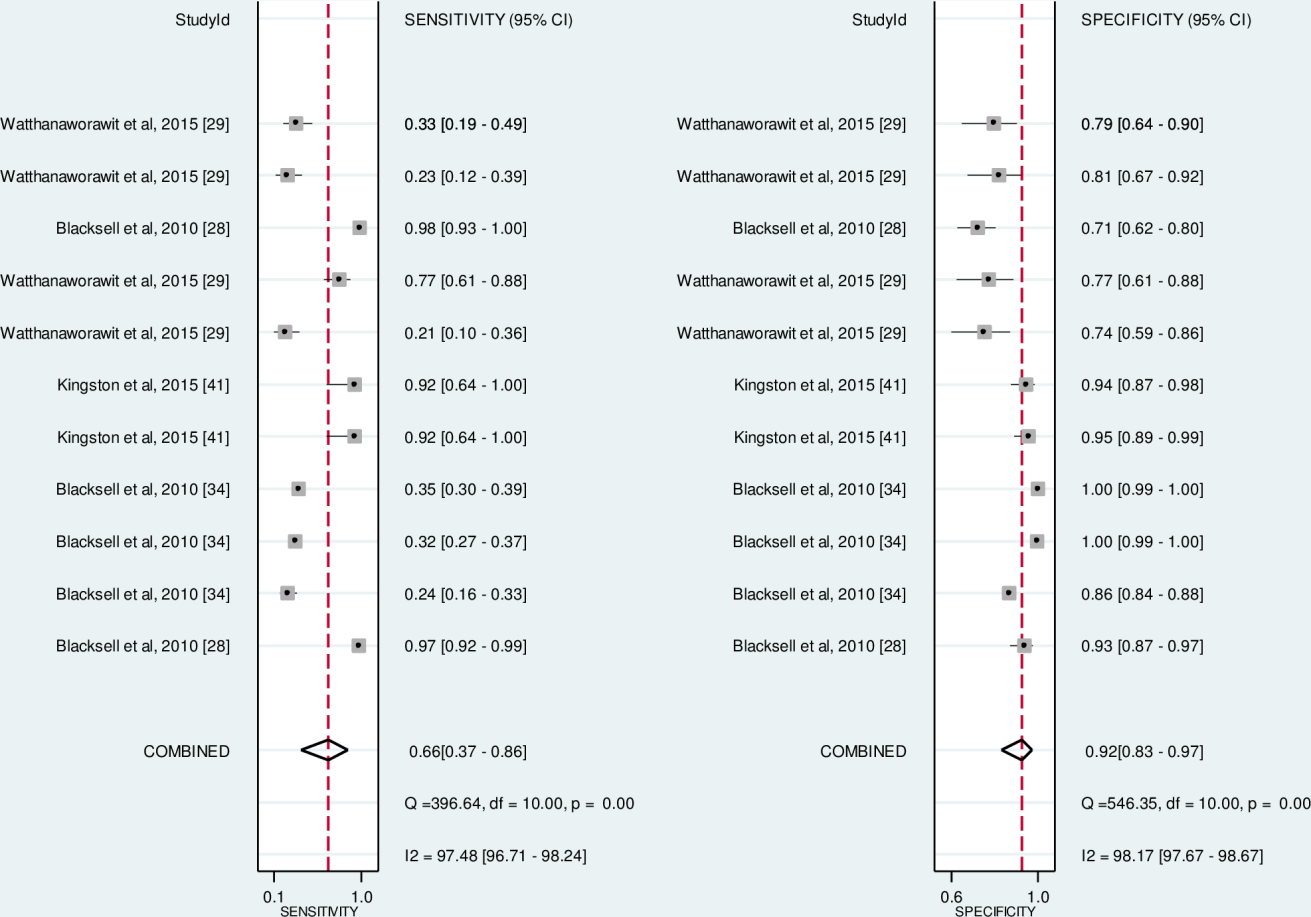
Forest plot of sensitivity and specificity of current commercially available POCT.

There were 11 data points extracted from four studies included in the meta-analysis (Fig 5). In the resulting forest plot (Fig 5), the top three data were extracted from studies assessing total antibody ICT (Blacksell et al, 2010 and Watthanaworawit et al, 2015) [28,29]. The rest of the 2 by 2 table data were extracted from studies assessing IgM ICT diagnostic performance. The pooled sensitivity and specificity were 66.0% (95% CI 0.37–0.86) and 92.0% (95% CI 0.83–0.97), respectively. The overall Chi-square heterogeneity statistics showed significant heterogeneity (p < 0.001). There is a high degree of heterogeneity present (I^2^ value = 97.48%, 95% CI 96.71–98.24 for sensitivity and I^2^ value = 98.17%, 95% CI 97.67–98.67 for specificity). Meta-regression on several covariates was performed in an attempt to explain this heterogeneity. Significant heterogeneity was observed for total number of samples (p = 0.01), study design (whether using case-control design or not, p = 0.01), blinding during index test interpretation (p = 0.02), and QUADAS-2 score (p = 0.01). No significant heterogeneity was observed for the blinding during reference test interpretation (p = 0.21) and antibody target detection of the tests (p = 0.22). All of these studies used IFA as their reference standard, except Blacksell et al, 2010 which used IFA with the addition of PCR and culture [28]. None of the meta-analysed studies used an IFA cut off lower than 1:400 as the reference comparator.

## Discussion

There were 31 relevant articles included in this review. Almost half of the included articles were of moderate quality. The meta-analysis showed moderately low pooled sensitivity and good specificity of the current commercially available scrub typhus POCT. However, the studies were heterogeneous with the I^2^ value indicating a high degree of heterogeneity. Hence, the pooled sensitivity and specificity value needs to be interpreted with caution.

The systematic review and meta-analysis highlighted the methodological and clinical heterogeneity across scrub typhus POCT diagnostic accuracy studies. These differences made it difficult to pool results and compare studies. Meta-regression for other covariates of interest (e.g. sample collection timing) could not be performed because of limited information presented in the original articles.

Almost a quarter of the responses gathered in the main seven questions on QUADAS-2 quality assessment were ‘unclear’. Although we did not assess the quality of reporting in this review, this finding indicates that the quality of reporting in the included studies is still arguably poor. Poorly conducted and controlled diagnostic accuracy studies are a waste of time, resources, and effort; moreover, if research is not accurately reported, it can hinder critical appraisal, replication, and meta-analysis of studies [30]. The launch of reporting guidelines such as Standards for Reporting Diagnostic Accuracy (STARD) and PRISMA is a starting point in improving the quality of reporting, although they are not applied as much as they should be [30,31]. Besides adhering to reporting guidelines, regulations should be adapted to incentivise better and more complete reporting [31]. Creating a reporting infrastructure and building the capability of both authors and reviewers are also necessary to encourage better reporting [31].

Approximately two thirds of the included studies used case-control design. Compared with studies that recruit patients consecutively, case-control study design (evaluating index test in separate diseased population and control group) overestimates diagnostic performance [32]. Therefore, the results presented need to be interpreted with caution.

Aside from LAMP assay, all of the other POCTs reviewed here relied on antibody detection. Serological diagnosis is problematic in several ways. First, the primary serum collection may not contain sufficient antibodies since it takes time for antibodies to increase to a detectable level creating a “false negative” result. Second, in endemic populations with significant background immunity, an appropriate cut-off needs to be established to ensure accurate diagnosis otherwise there is the possibility of “false positives”. Furthermore the issues regarding the selection of IgM as opposed to whole antibody is very much dependent on the situation. Conventional thought is that whole antibodies may give higher number of “false positive” results in endemic situations due to the presence of residual IgG from previous scrub typhus infections as was the case with the AccessBio ICTs tested in Thailand and Laos [19,28]. These shortcomings of serology highlight the need to develop alternative diagnostic strategies.

Although being pooled from heterogeneous studies, the cumulative specificity confidence interval was above 80.0%. This indicates that commercially available ICTs have value in “ruling in” for the diagnosis of scrub typhus. However, it is difficult to draw conclusions with confidence based on the currently available evidence given the high degree of heterogeneity amongst the studies.

Another important consideration when performing a diagnostic accuracy study is the choice of reference comparator. Often the IFA, the serological “gold standard”, is selected. However, this test is far from perfect. IFA result interpretation is subjective and there remains a lack of consensus on the optimum positivity cut-off. A cut-off is often adopted without sufficient local evidence, potentially resulting in incorrect diagnostic accuracy measures for the diagnostic under test [15]. To evaluate the true accuracy of IFA, Bayesian latent class models (LCM) have been used. The models showed that the IFA IgM has sensitivity and specificity of 70.0% and 83.8%, respectively; therefore suggesting it to be unfit as a reference standard [12]. An alternative reference comparator is the composite Scrub Typhus Infection Criteria (STIC), which have been proposed as a more robust method to diagnose scrub typhus with more confidence, by including a panel of parameters with high specificity [20]. The panel includes: (i) isolation of *O*. *tsutsugamushi*, (ii) at least two positive out of the three PCR assays targeting the 56kDa, 47kDa, and groEL genes, (iii) admission IFA IgM titre of ≥1:12,800, (iv) 4-fold rise in IgM titre from paired samples [12,19,20]. At least one of these criteria needs to be fulfilled for a positive scrub typhus diagnosis. However the Bayesian LCM also showed that STIC’s sensitivity and specificity are less than optimal for a reference test (90.5% and 82.5%, respectively).

This study has several limitations. First, the search was performed in one database, and in English only which might have resulted in non-inclusion of relevant articles. Second, the article inclusion and quality assessment were completed primarily by one person, though we attempted to decrease the risk of bias by routine discussion of contentious studies amongst the authors. Third, several studies did not present all of the parameters that we wished to extract, hence limiting the meta-analysis, as we could not include all relevant papers due to the limited information presented in the papers.

In reality, there is only a small number of scrub typhus POCTs that are commercially available. The Panbio POCTs are no longer available and therefore the market is dominated by a few companies including InBios, Standard Diagnostics and AccessBio. Selecting the most appropriate POCT is very much dependant on a few key factors such as availability of product locally, the price and the shelflife. In this study we have not examined these local practical aspects that should be considered when selecting POCTs.

In the absence of robust POCTs, the presence of an eschar can be a valuable clinical sign strongly suggesting a diagnosis of scrub typhus. Although its reported presence is very variable, it is still regarded as pathognomonic. An eschar may go unnoticed by the patient since it is painless, often does not itch, can be small, and looks similar to post trauma scab. In addition, it may be located in a concealed area, such as the perineum or under the breasts. This emphasises the importance of performing a thorough physical examination.

Treating patients empirically based on the pathogen pattern in an area is common practice, but bears the risk of unnecessary or inappropriate treatment (with the attendant risks of side effects) and promotion of antimicrobial resistance. POCTs can play an important role in reducing the number of patients treated empirically and increasing the proportion of patients treated appropriately.

There is an urgent need to develop an affordable and accurate POCT. However, if POCTs still rely on serological measures only, they might not be able to provide diagnosis in time to inform treatment. Future research should also be directed towards developing new antigen-based tests to improve diagnostic accuracy in the early period of disease.

## Supporting information

S1 Dataset QUADAS-2 scores. (XLSX)

S2 Dataset QUADAS-2 sub-question. (XLSX)

S3 Dataset Extracted data. (XLSX)

S1 Checklist PRISMA Checklist. (DOC)
